# Posterior Segment Ocular Trauma: Timing and Indications for Vitrectomy

**DOI:** 10.1155/2017/5250924

**Published:** 2017-10-26

**Authors:** Robert Rejdak, Anselm G. Juenemann, Sundaram Natarajan

**Affiliations:** ^1^Department of General Ophthalmology, Medical University in Lublin, Lublin, Poland; ^2^University Eye Hospital, Rostock, Germany; ^3^Aditya Jyot Eye Hospital, Mumbai, India

Posterior segment ocular trauma is still an important cause of visual loss and disability in working-age population. With modern surgical approaches, many eyes can be salvaged with retention of vision. Both open (penetration injury, intraocular foreign body, and eye rupture) and closed (retinal detachment, vitreous hemorrhage, and macular hole) eye injuries may be indications for vitrectomy. Timing of vitrectomy is still a matter of discussion and depends on surgeon's experience and capabilities.

The most urgent indication for vitrectomy is endophthalmitis. Y. Sheng et al. performed a systematic review of the Chinese literature on pediatric posttraumatic endophthalmitis and described the epidemiology, management, causative organisms, and visual acuity outcomes of the reported cases in twenty years. They found that *Staphylococcus epidermidis* was the most common isolated organism and the use of a disposable syringe needle was the most common cause of ocular injuries in pediatric posttraumatic endophthalmitis in China.

Another indication for vitrectomy is intraocular foreign body. In this issue, D. A. Guevara-Villarreal and P. J. Rodríguez-Valdés described extraction surgical techniques, timing, and indications for vitrectomy due to intraocular foreign body. Potential advantages of immediate IOFB removal include a possible decrease in risk of endophthalmitis, a decrease in the rate of proliferative vitreoretinopathy (PVR), and a single procedure for the patient. Early surgery was not significantly associated with greater visual improvement but had a significant impact on the development of posttraumatic endophthalmitis. The authors conclude that the most important factor at the time to perform IOFB extraction is the experience of the surgeon.

G. Sborgia et al. evaluated the safety and outcomes in patients with open eye injury with retained foreign body that underwent early 25-gauge vitrectomy. They concluded that pars plana vitrectomy is considered the most effective and safest approach for the removal of retained ocular foreign bodies and repair of retinal injuries correlated. Advances in small-gauge (25-gauge or 27-gauge) vitrectomy instrumentation as well as surgical techniques have increased indications for complex cases.

R. Rejdak et al. evaluated visual and safety outcomes of 23-gauge pars plana vitrectomy with application of perfluorocarbon liquid for intraoperative protection of the macula during intraocular foreign body removal. They have not observed any iatrogenic injury of the macula during PPV for metallic IOFB removal with PFCL use in our group of 42 patients. However, falling of IOFB was reported in 3 patients without retinal injury within the macula. Thus, the macula can be a shield from IOFB drop during its extraction by reduction of impact force of falling IOFB or by deflecting its trajectory toward the peripheral retina.

Traumatic retinal detachment is another indication for vitrectomy in posterior ocular trauma. There are currently no evidence-based guidelines on the appropriate time to perform vitreoretinal surgery to repair a traumatic retinal detachment. Early intervention, within seven days of the inciting trauma, may decrease proliferative vitreoretinopathy and postoperative endophthalmitis. Later intervention may yield a reduced risk of inflammation and hemorrhage, particularly in cases of concomitant open globe injuries. M. Orban et al. reviewed the literature on the management of retinal detachments associated with ocular trauma from the years 2006 to 2016. Preliminary data indicate that endophthalmitis rates may be lower when early vitreoretinal surgery is performed.

K. Nowomiejska et al. evaluated functional and anatomical results of pars plana vitrectomy in the retinal detachment followed by severe eye trauma in the retrospective analysis of medical records of forty-one consecutive patients treated with 23-gauge PPV due to traumatic retinal detachment. They concluded that among analysed pre- and intra- and postoperative factors, absence of PVR, postoperative retinal attachment, and silicone oil as a tamponade were related to significantly improved visual acuity.

W. Liu et al. reviewed current management of traumatic macular holes and they state that it is not a rare clinical condition, especially in young population. Although TMH commonly occurs in closed globe injuries caused by blunt ocular trauma, the mechanism of the hole formation remains controversial. Due to the relatively high rate of spontaneous closure of traumatic macular hole, it has been suggested that adult patients may be observed for 3 to 6 months after the hole formation, especially in young patients with small holes, good visual acuity, and posterior vitreous adhesion to the hole edges. Surgery may be recommended earlier for pediatric patients to prevent amblyopia, depending on the age of the child. Modern vitrectomy surgery plays an important role in the treatment of traumatic macular hole, although the functional outcomes may be compromised by the concomitant retinal pathologies.

H. H. Ghoraba et al. reported the difference in either anatomical or functional outcome of vitreoretinal intervention in cases of gunshot perforating eye injury if done 2–4 weeks or after the 4th week after the original trauma. Patients were treated with pars plana vitrectomy and silicon oil. There was no statistically significant difference between the two groups in either anatomical or functional results. The authors conclude that the visual outcome after gunshot perforating eye injury depends on the site of entry and exit (the route of gunshot).

In summary, this issue includes different surgical approaches and will provide valuable clinical information that should be helpful in clinical practice for vitreoretinal surgeons. This will improve management of patients after severe eye trauma with posttraumatic endophthalmitis, intraocular foreign body, posttraumatic retinal detachment, and traumatic macular hole.

*Robert Rejdak* *Anselm G. Juenemann* *Sundaram Natarajan*

